# The Potential of Low-Cost IoT-Enabled Agrometeorological Stations: A Systematic Review

**DOI:** 10.3390/s25196020

**Published:** 2025-10-01

**Authors:** Christa M. Al Kalaany, Hilda N. Kimaita, Ahmed A. Abdelmoneim, Roula Khadra, Bilal Derardja, Giovana Dragonetti

**Affiliations:** The Mediterranean Agronomic Institute, CIHEAM Bari, Valenzano, 70010 Bari, Italy; kly@iamb.it (C.M.A.K.); kimaitahilda@gmail.com (H.N.K.); ayoub@iamb.it (A.A.A.); derardja@iamb.it (B.D.); dragonetti@iamb.it (G.D.)

**Keywords:** agrometeorological station, IoT, environmental monitoring, sensors, wireless communication

## Abstract

The integration of Internet of Things (IoT) technologies in agriculture has facilitated real-time environmental monitoring, with low-cost IoT-enabled agrometeorological stations emerging as a valuable tool for climate-smart farming. This systematic review examines low-cost IoT-based weather stations by analyzing their hardware and software components and assessing their potential in comparison to conventional weather stations. It emphasizes their contribution to improving climate resilience, facilitating data-driven decision-making, and expanding access to weather data in resource-constrained environments. The analysis revealed widespread adoption of ESP32 microcontrollers, favored for its affordability and modularity, as well as increasing use of communication protocols like LoRa and Wi-Fi due to their balance of range, power efficiency, and scalability. Sensor integration largely focused on core parameters such as air temperature, relative humidity, soil moisture, and rainfall supporting climate-smart irrigation, disease risk modeling, and microclimate management. Studies highlighted the importance of usability and adaptability through modular hardware and open-source platforms. Additionally, scalability was demonstrated through community-level and multi-station deployments. Despite their promise, challenges persist regarding sensor calibration, data interoperability, and long-term field validation. Future research should explore the integration of edge computing, adaptive analytics, and standardization protocols to further enhance the reliability and functionality of IoT-enabled agrometeorological systems.

## 1. Introduction

Agriculture is highly sensitive to climate change, as unprecedented weather patterns along with an increase in extreme events such as droughts and floods pose significant threats to crop yields and overall food security [1]. Climate change disrupts growing seasons, reduces yields, promotes the spread of pests and diseases, and imposes heat and water stress on crops and livestock threatening global agricultural sustainability [2,3]. Conventional weather stations and satellite systems have provided valuable insights, they often face limitations including high costs, delayed updates, a lack of tailored and localized information, limited user capacity and limited spatial resolution [4]. This presents challenges for smallholder farmers, who make up a significant portion of the global agricultural workforce and often lack access to localized, timely weather data. In response, affordable IoT-enabled agrometeorological stations offer a scalable and accessible solution [5]. This underscores the need for more accessible, real-time meteorological solutions to support climate-resilient agriculture [1,6].

In this context, access to agrometeorological data including temperature, humidity, rainfall, wind speed, and solar radiation becomes critical for informed decision-making [7]. Timely and localized weather information enables farmers to better plan irrigation, fertilization, pest control, and harvesting, thereby improving yields and resource efficiency [8,9].

In recent years, the adoption of digital technologies in agricultural management has led to the development of smart farming, also known as digital agriculture. Central to this advancement is the Internet of Things (IoT), which connects physical devices such as sensors, microcontrollers, and weather stations to facilitate real-time data collection and communication [10]. This progress enables climate-smart agriculture, utilizing data analytics to optimize resource inputs such as water, fertilizers, and pesticides, thereby enhancing efficiency and reducing waste [11].

A vital element of this ecosystem is the IoT-enabled agrometeorological station an advanced weather monitoring system equipped with interconnected sensors that measure parameters including temperature, humidity, rainfall, wind speed, and solar radiation [12]. These weather data systems transmit data in real time to cloud platforms or mobile devices, supporting continuous, automated monitoring. Unlike conventional weather stations, which often depend on manual data collection or limited telemetry [13].

For example, ref. [14] stated that smart irrigation systems can automatically adjust water application based on real-time soil and weather data, while connected weather stations deliver hyper-local forecasts that inform daily farming practices. These innovations not only improve productivity and operational efficiency but also enhance resilience to climate-related challenges.

By reducing barriers to adoption, ref. [15] declared that these systems enable inclusive data collection in rural and remote areas, empowering smallholder farmers to participate more actively in digital farming initiatives. Enhanced access to accurate weather information can strengthen farm resilience to climate variability, support informed management decisions, and contribute to food security and sustainable development within vulnerable communities [16].

This systematic review examines low-cost IoT-enabled agrometeorological stations, applying criteria that prioritize affordability, IoT integration, empirical evidence, comprehensive system components, and relevance to climate-smart agriculture. By focusing on low-cost solutions, the review aims to enhance accessibility for smallholder farmers. The inclusion of studies utilizing IoT technologies is crucial for understanding their role in real-time data collection and informed decision-making in agriculture. Empirical findings from real-world contexts ensure practical assessments of system effectiveness and challenges. Moreover, to maintain rigor and comparability, the review utilized the SPIDER framework (Sample, Phenomenon of Interest, Design, Evaluation, Research type) for clearly defined inclusion and exclusion criteria. This approach allows for a focused analysis of studies relevant to low-cost IoT agrometeorological stations in agricultural settings while excluding conceptual papers and high-cost systems not applicable to smallholder contexts. By justifying the review scope in this manner, the analysis provides a transparent foundation for evaluating the potential of these innovative systems compared to conventional weather stations.

In the below Table 1, the systematic reviews concerning weather station technologies, reveal considerable improvements facilitated by the integration of Internet of Things (IoT), Artificial Intelligence (AI), Machine Learning (ML), Wireless Sensor Networks (WSN), and cloud computing.

These technological innovations have substantially enhanced the precision, operational efficiency, and real-time accessibility of meteorological data, offering critical support to sectors such as agriculture, disaster risk management, transportation, and public health. Refs. [14,19,23] particularly emphasized the utility of cost-effective, Arduino-based IoT systems, which enable remote environmental monitoring, continuous data logging, and real-time visualization capabilities that are especially beneficial in rural and re-source-constrained contexts.

Despite these advancements, the literature identifies persistent challenges that limit broader adoption and effectiveness. Ref. [17] highlights data reliability issues stemming from human error and inadequate sensor calibration. Ref. [18] pointed to limitations in the predictive accuracy of AI-based algorithms. Ref. [19] noted technical constraints, including dependency on stable inter-net connectivity, limited sensor functionalities, and insufficient data storage capacity. Ref. [20] discussed the limited availability of localized, context-specific weather forecasts, while [20,21] identified infrastructural deficiencies and inadequate technical training in low-income settings. Furthermore, ref. [21] highlighted a lack of standardization and fragmentation in research efforts, particularly in health-related weather monitoring applications. Ref. [22] revealed thematic gaps, such as insufficient attention to high-value crops and a lack of focus on mitigation strategies and policy integration in the context of extreme weather events.

However, several recurring limitations constrain up-scaling data quality, standardization, and deployment as mentioned across the articles. Refs. [17,18,22] showed that up-scaling and external validity are limited due to context specific data or region focused studies, often with insufficient benchmarks for broader application. Refs. [14,21,23] noted that data quality, calibration, and validation are hampered by calibration errors, maintenance needs, environmental influences on accuracy, and gaps in long-term validation. Standardization and interoperability are hindered by a lack of standardization and calibration comparability, making cross-site comparisons and large-scale deployment difficult [14,21,23]. Uncertainty quantification and model robustness are frequently inadequate or absent, complicating validation under extreme or real-time conditions [18,21]. Additionally, refs. [19,21,23] highlighted that cost, accessibility, and scalability remain major barriers, with high costs and limited accessibility, plus concerns about scalability and deploy ability at scale. Data governance, privacy, and security concerns are raised as barriers to adoption [21]. Finally, ref. [20] mentioned that capacity-building and uptake are essential to translate forecasts and sensor data into actionable use across sectors, underscoring the need for training, tool development, and stakeholder engagement.

Consequently, this systematic review aims to analyze the common hardware and software components utilized in these systems including sensors, microcontrollers, and communication technologies to understand prevailing design patterns and technological choices. Furthermore, the study will evaluate the comparative potential of low-cost IoT- based stations relative to conventional weather stations. It also emphasizes their contribution to improving climate resilience, facilitating data-driven decision-making, and expanding access to weather data in resource-constrained environments key factors in supporting agronomic planning and climate-smart agriculture. By doing so, the research aims to contribute to the growing body of knowledge on climate-smart agriculture, offering actionable insights that can drive the adoption of technology in rural settings and increasing climate variability.

## 2. Materials and Methods

This systematic review was conducted in accordance with the Preferred Reporting Items for Systematic Reviews and Meta-Analyses (PRISMA) guidelines.

### 2.1. Research Strategy

To ensure comprehensive coverage, two key databases were selected: Scopus and Web of Science, known for their extensive indexing of peer-reviewed literature. These sources were chosen for their accessibility and their strong representation of publications related to agrometeorological stations, IoT, environmental monitoring and wireless communication. On 4 July 2025, the search terms were organized into keyword groups and combined using the Boolean expression (set1 AND set2 AND set3) to target relevant studies and filter out unrelated topics:

1. (“Meteorological station” OR “Weather station”) AND (“IoT”) AND (“Precision Agriculture”)
2. (“Meteorological station” OR “Weather station”) AND (“IoT”) AND (“Agriculture”)
3. (Agrometeorological station OR “Weather station”) AND (“IoT”)
4. (“IoT” AND “smart farming” OR “digital agriculture”) AND (“weather data” OR “environmental monitoring”)
5. (“Low-cost weather stations” OR “affordable IoT solutions”) AND (“climate resilience” OR “sustainable agriculture”)

These queries aim to capture a wide range of aspects related to IoT technology in agricultural contexts, focusing on the incorporation of affordable solutions, the significance of wireless sensor networks, and the effects of real-time monitoring on farming practices. This strategy facilitates a more thorough investigation into the increasing implementation of advanced technologies in weather monitoring.

### 2.2. Study Selection

**Figure 1:**
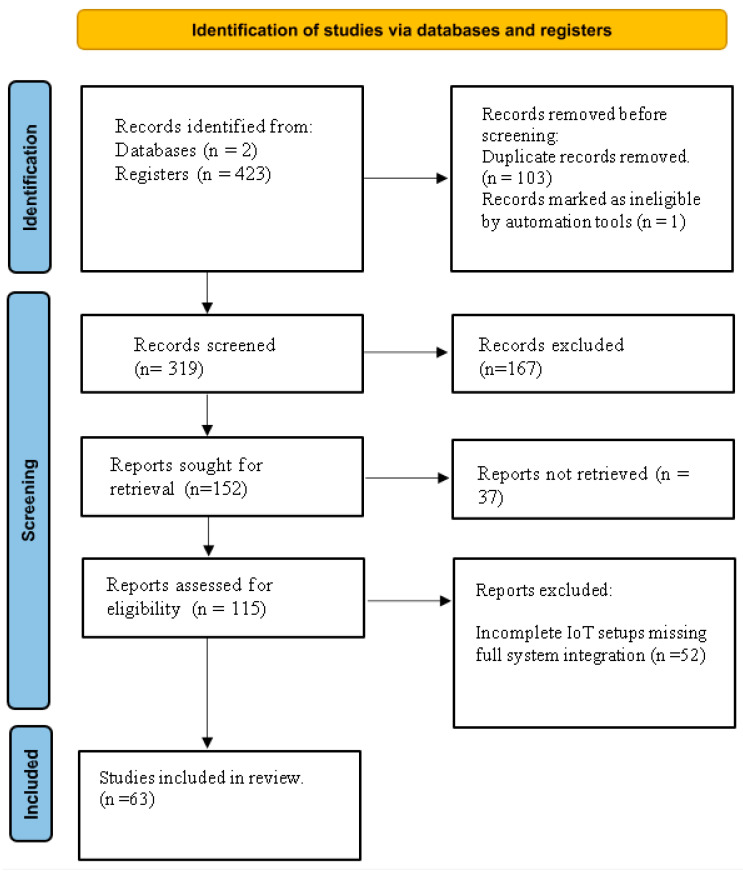
The screening process PRISMA chart.

The initial search yielded a total of 423 articles, distributed across the databases as follows: 197 from Web of Science, and 226 from Scopus. A visual overview of the selection process is provided in Figure 1. After removing duplicates, 320 records remained for title and abstract screening. This screening process resulted in the exclusion of 167 articles that did not meet the specified inclusion criteria, leaving 115 studies eligible for full-text review. Following a comprehensive evaluation, 63 articles were selected for final inclusion based on their relevance to IoT, wireless communication, and agrometeorological stations.

Additionally, this review included peer-reviewed journal and articles between 2015 and 2025. Only English-language publications were considered. Theses, patents, and non-English works were excluded.

Analysis of the publication years indicates that research activity peaked in 2021. However, there was a subsequent decline in 2022, and a moderate resurgence in 2023 and 2024, demonstrating evolving interest in this area.

Moreover, in selecting the cited sources for this review, we carefully considered the number of citations to ensure the relevance and significance of the included articles. We prioritized studies published in high-impact journals with significant citation counts, as these metrics reflect the influence and credibility of the research within the academic community. This approach ensures that our review is grounded in the most recognized and impactful literature related to low-cost IoT-enabled agrometeorological stations.

### 2.3. Inclusion and Exclusion Criteria

Studies were required to meet the following inclusion criteria to be considered for this review: (1) Focused on low-cost agrometeorological stations that incorporate Internet of Things (IoT) technologies for collecting environmental or agricultural data including: temperature, humidity, soil moisture, rainfall, and wind speed; (2) Eligible studies needed to describe the design, implementation, or deployment of these platforms in real-world agricultural or rural settings and discuss the integration of IoT components such as microcontrollers, sensors, wireless communication modules, and cloud or data platforms; (3) Studies that presented empirical findings, case studies, or practical evaluations regarding system performance, scalability, cost-effectiveness, or im-pact on end-users in agricultural contexts.

However, studies were excluded if they were: (1) Conceptual papers, or purely theoretical discussions without empirical validation; (2) Research focused on high-cost or industrial-grade meteorological stations without addressing affordability or accessibility for smallholder or resource-limited farmers was also excluded; (3) Studies that did not include IoT elements in the system design, or that solely addressed sensor development, communication protocols, or data analytics without linking them to a complete agrometeorological station, were not considered. The eligibility criteria were formulated based on the Sample, Phenomenon of Interest, Design, Evaluation, Re-search Type (SPIDER) framework [24], as detailed in Table 2.

### 2.4. Screening and Data Extraction

Following the search of the specified databases and the removal of duplicate entries, the authors conducted an initial screening of titles and abstracts to exclude irrelevant studies. The remaining articles were subjected to full-text review, and those meeting the inclusion criteria were incorporated into the systematic review. A standardized data extraction protocol was developed to organize detailed information from each study to ensure consistency and thoroughness across all selected studies.

Key information was systematically documented, including the authorship and publication year, technological specifications and system components (sensor types and their environmental parameters, communication protocols, power sources, hardware configuration and modes of data access) and the intended agricultural application for each station. When available, system cost estimates were recorded to evaluate affordability. Additionally, to ensure a comprehensive review, performance metrics such as accuracy, reliability, and sensor calibration outcomes were extracted, along with any limitations reported by the studies. This protocol facilitated a comprehensive comparative analysis of technological components, operational contexts, and agronomic relevance across the reviewed literature.

## 3. Results and Discussion

### 3.1. Hardware Components

#### 3.1.1. Microcontroller Platforms

**Figure 2:**
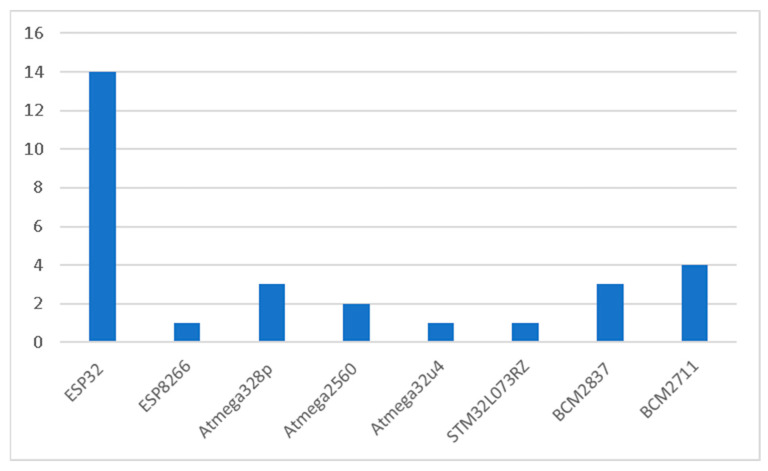
The most used microcontrollers.

As shown in Figure 2, the distribution of microcontroller families across the reviewed studies highlights the technological preferences guiding the design of low-cost agrometeorological stations. Among these, the ESP32 family stands out as the most widely adopted. Its dominance is explained by its advanced features, including dual-core processing, built-in Wi-Fi and Bluetooth connectivity, and efficient power management [25]. These attributes make ESP32 highly suited for Internet of Things (IoT) applications in agriculture, where seamless sensor integration and wireless data transmission are essential [26]. Furthermore, its processing capability enables near real-time data handling, which is particularly valuable for tasks such as automated irrigation and dynamic environmental monitoring [27,28]. A limitation, however, is that extended wireless communication can increase energy consumption, which poses challenges for long-term battery-powered systems [29].

The ESP8266 family also appears in the reviewed designs, though less frequently. As a predecessor to ESP32, ESP8266 provides basic Wi-Fi connectivity at a very low cost, making it an attractive option for simple IoT applications [30]. While limited in terms of memory and processing power, it remains a useful alternative in scenarios where affordability and basic connectivity are prioritized over computational capacity.

Atmega microcontrollers (Atmega328p, Atmega2560, Atmega32u4) are another key group in agrometeorological applications. Their popularity stems from low cost, robustness, and ease of integration with sensors. The Atmega328p is particularly valued for its balance between simplicity and flexibility, while the Atmega2560 offers expanded memory and input/output capacity for more complex systems. The Atmega32u4, on the other hand, is often selected when native USB communication is required. Despite their accessibility, Atmega microcontrollers are limited by relatively low processing power, which constrains their performance in applications requiring advanced analytics or handling of high-resolution datasets [9].

In addition, STM32 microcontrollers, such as the STM32L073RZ, provide an alternative platform for applications requiring low-power ARM Cortex architectures. These microcontrollers are known for their energy efficiency and reliability, often making them suitable for industrial or professional-grade deployments where system stability and long-term performance are crucial.

Finally, the Broadcom BCM2837 and BCM2711 families which power the Raspberry Pi 3 and Raspberry Pi 4, respectively, are included in several systems requiring greater computational capabilities. Unlike traditional microcontrollers, these system-on-chips (SoCs) enable the execution of more advanced tasks, such as local data processing, machine learning, and complex visualization. Their role in agrometeorological stations is typically associated with edge computing, where real-time analytics are carried out locally rather than in the cloud [31,32]. However, these platforms also require significantly more energy compared to microcontrollers like Atmega or ESP families, which can be a limiting factor in remote or energy-constrained agricultural environments.

Overall, the choice of microcontroller family reflects a balance between cost, processing power, energy efficiency, and communication needs. ESP32 dominates due to its strong combination of wireless connectivity and processing capability, while Atmega families continue to be favored for their affordability and simplicity [33]. Raspberry Pi families (BCM2837 and BCM2711) are chosen where advanced analytics and edge-based computation are needed, whereas STM32 and ESP8266 fill niche roles in low-power or cost-sensitive deployments. As emphasized by [34,35], the hardware platform significantly affects scalability, reliability, and long-term maintenance of agrometeorological stations, underscoring the importance of carefully aligning microcontroller selection with application-specific requirements.

#### 3.1.2. Development Boards

**Figure 3:**
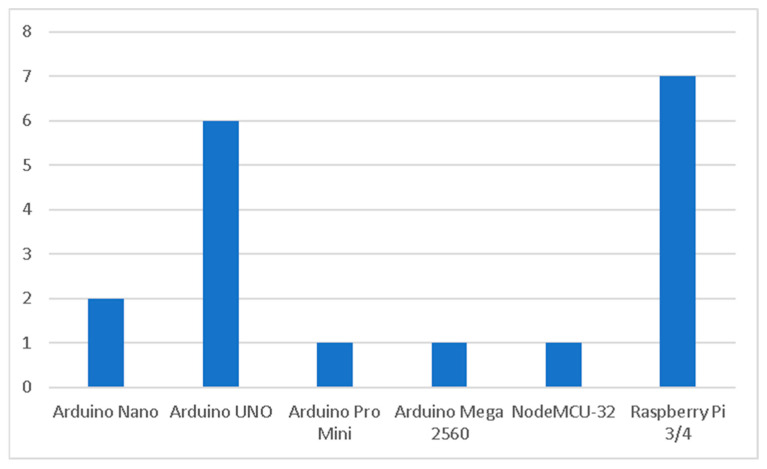
The Development boards used.

As shown in Figure 3, the reviewed studies reveals that the selection of development boards plays a pivotal role in shaping the performance and adaptability of low-cost agrometeorological stations. Among them, Arduino-based boards such as Arduino Nano, Arduino UNO, Arduino Pro Mini, and Arduino Mega 2560 are widely adopted due to their affordability, modular architecture, and ease of programming [11]. Costing typically between $20 and $40, these boards are accessible for smallholder farmers and researchers with limited resources [27,36]. In addition, their strong open-source ecosystem and large community provide extensive libraries and tutorials, facilitating rapid sensor integration and system customization for applications such as environmental monitoring, soil sensing, and basic irrigation scheduling. However, studies have noted that these boards can struggle with advanced computational tasks, given their relatively limited memory and processing power [9].

In contrast, Raspberry Pi boards (models 3 and 4) are selected when higher computational capacity is required. They differ from simpler boards by supporting full operating systems, enabling local execution of advanced analytics, machine learning models, and real-time visualization of environmental data [31,37]. Previous research has shown that Raspberry Pi can serve as a coordinator hub in distributed wireless sensor networks, effectively managing data aggregation, decision-making, and irrigation control [32,38]. Despite their versatility, Raspberry Pi platforms are more energy-intensive and costlier than Arduino boards, which can present barriers for long-term use in low-resource or off-grid agricultural settings.

Specialized platforms such as NodeMCU-32, and Heltec LoRa ESP32 microcontrollers also appear in certain studies, particularly where wireless communication, robustness, or autonomy is prioritized. For instance, NodeMCU-32 and Heltec LoRa ESP3 facilitate seamless IoT connectivity, enabling long-range communication and integration with cloud services. Such platforms are particularly valuable in energy-autonomous stations designed for remote environments [39], although their higher complexity and setup requirements may present challenges for small-scale users.

The analysis confirms that development board selection reflects a balance between simplicity and affordability (Arduino), computational sophistication (Raspberry Pi), and specialized autonomy (NodeMCU-32, Heltec LoRa ESP3, and marine-grade gateways).

#### 3.1.3. Communication Technologies

The choice of communication technologies in the reviewed studies reflects a strategic balance between range, energy efficiency, and data reliability. To ensure clarity, these technologies can be classified according to their functional layer in the IoT communication stack.

a- Physical and MAC Layer Technologies

At the physical and medium access level, several options were reported. Wired connections such as Ethernet appear less frequently in agrometeorological stations but remain relevant in controlled environments, where stable and high-speed connections are required [40]. Their main advantage is reliability, though they lack the flexibility needed for large agricultural fields [41].

Among wireless solutions, Wi-Fi (IEEE 802.11 PHY/MAC) is widely employed due to its availability and compatibility with common development boards [42,43]. It is well suited for peri-urban or pilot-scale applications where existing infrastructure is available [27,31]. However, its short range and high-power consumption constrain its use in remote or energy-limited deployments [44].

LoRa (PHY) has emerged as a leading solution for long-range, low-power transmission. It enables communication across 2–10 km with very low energy consumption (10–50 mW) [45,46]. When deployed with LoRaWAN (MAC/network layer), it supports scalable multi-node networks and coordinated data management [40,47]. For example, LoRaWAN implementations that transmit only threshold-based data significantly reduce energy use while maintaining relevance [47].

Cellular technologies (GSM, GPRS) are adopted in scenarios where Wi-Fi or LoRaWAN coverage is unavailable. They provide extensive coverage and higher data rates (40–200 kbps) but at the expense of higher energy consumption (200–300 mW) and ongoing data costs, which may limit their suitability for smallholder farmers [34,48].

b- Network and Transport Protocols

Above the physical/MAC layer, network and messaging protocols facilitate device-to-server communication. MQTT is the most widely adopted lightweight publish–subscribe protocol in the reviewed systems. With low bandwidth requirements (≈10 kB per message), it efficiently supports multi-sensor networks and farm dashboards [11,49]. Its limitations are linked to dependency on stable internet connectivity, which is not always available in rural contexts. HTTP-based APIs were also used, particularly in systems requiring standardized web integration [49].

c- Application-Layer and Cloud Integration Services

Several studies reported the use of ThingSpeak and similar dashboards. These are not communication technologies but rather cloud data platforms that provide visualization, storage, and analytics [50]. They complement underlying protocols by offering user-friendly interfaces for farmers and researchers, thereby improving usability and decision support [51,52].

The comparison across layers demonstrates that no single communication technology can fully address the diverse requirements of agrometeorological stations. Physical/MAC solutions such as Wi-Fi and cellular technologies are advantageous for real-time control and high data rates but are constrained by range and energy demands, whereas LoRa/LoRaWAN is optimal for wide-area, low-power monitoring but sacrifices bandwidth. At the network and transport layer, protocols such as MQTT and HTTP enable interoperability and lightweight messaging, ensuring efficient data delivery across heterogeneous deployments. Finally, cloud integration services such as ThingSpeak extend these communication frameworks by providing storage, visualization, and decision-support tools, which are essential for user adoption and actionable insights. Taken together, these layers are complementary rather than competitive: effective agrometeorological systems often rely on hybrid architectures that combine short-range and long-range physical links with efficient transport protocols and cloud platforms. This layered approach maximizes scalability, energy efficiency, and data reliability, in line with the need for interoperable and standardized IoT solutions in agriculture.

#### 3.1.4. Sensor Types and Environmental Parameters

**Figure 4:**
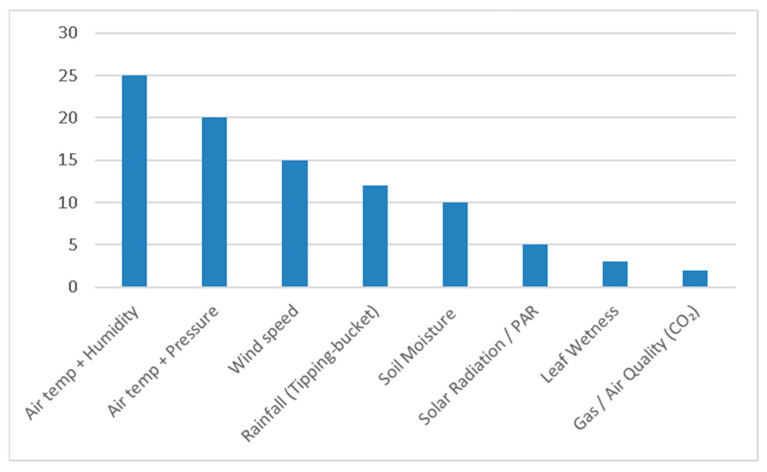
Most used sensors in Low-cost weather stations.

Environmental sensing in the reviewed studies was predominantly structured around standard agrometeorological parameters, reflecting the need for accurate weather monitoring to support climate-smart agricultural practices, as shown in Figure 4. The most common parameter bundles included air temperature with relative humidity, and temperature with barometric pressure, owing to their interdependence in atmospheric compensation. These measurements are fundamental for weather forecasting, evapotranspiration estimation, and heat/frost risk assessment [30,31]. Sensors for these parameters were typically based on thermistors or resistance temperature detectors (RTDs) for temperature, capacitive sensors for humidity, and MEMS barometers for atmospheric pressure. Together, these bundled measurements form the backbone of localized weather monitoring, but they require regular calibration and compensation to maintain accuracy under variable environmental conditions.

Wind monitoring was also prominent, though approaches varied. Traditional configurations employ co-located cup anemometers and wind vanes, which provide scalar wind speed and directional bearing. In contrast, ultrasonic vector anemometers measure both wind speed and direction simultaneously as 2-D vectors, offering higher precision but at increased cost and power demand [53,54]. Wind speed measurements are vital for pesticide application, greenhouse ventilation, and microclimate management, where wind-driven evapotranspiration directly influences irrigation scheduling and crop water use.

Rainfall sensing was generally achieved through tipping-bucket pluviometers, valued for their simplicity and ability to provide real-time precipitation data. In studies such as [52,53] stated that these sensors support irrigation scheduling by preventing overwatering, although they may suffer from underestimation during high-intensity events or blockage by debris.

Soil moisture monitoring, though directly linked to precision irrigation, was less common compared to atmospheric sensing [54]. Technologies included time-domain reflectometry (TDR), frequency-domain reflectometry (FDR), and capacitive probes, often used in combination with temperature measurements to account for thermal effects on dielectric properties. Despite their agronomic importance, ref. [55] declared that adoption was limited by high costs (ranging from $1450 to several hundred euros) and the need for site specific calibration across heterogeneous soils. Nevertheless, studies have shown that integrating soil moisture data into decision-support systems significantly improves water productivity relative to weather-based irrigation alone [38].

Advanced sensing technologies were less frequently observed. Solar radiation can support crop growth modeling and evapotranspiration estimation, while leaf wetness sensors inform disease forecasting [33,56]. Despite their utility, these parameters are typically restricted to high-value cropping systems or precision agriculture research due to budget constraints. Similarly, gas (electrochemical CO_2_) and air quality sensors were mostly used in greenhouse-focused studies such as [30,57]. These provide insights into plant environment interactions but remain niche within broader agrometeorological deployments.

Overall, the reviewed literature indicates that sensor selection is strongly shaped by cost, ease of integration, and the intended agronomic application. Core atmospheric bundles provide a baseline for general weather monitoring, while underutilized soil and crop-focused sensors constrain the ability of these weather monitoring systems to support data-driven irrigation and crop-specific decision-making. As emphasized by [34,35], calibration against reference-grade weather stations is essential to improve the accuracy of low-cost rainfall and soil moisture sensors, which otherwise introduce systematic biases. A more holistic sensor suite that integrates atmospheric, soil, and crop parameters would substantially enhance the agronomic value of low-cost IoT agrometeorological stations.

### 3.2. Architecture of Low-Cost Agro-Monitoring Systems

The architecture of low-cost IoT-enabled agrometeorological stations typically follows a layered design comprising sensor nodes, processing units, communication modules, and data services. This modular organization ensures adaptability, scalability, and efficient monitoring across diverse agricultural environments.

The primary function of these systems is to monitor key agrometeorological parameters such as temperature, humidity, soil moisture, rainfall, and solar radiation. For instance, the smart solar-powered weather station developed by [56] demonstrates how renewable energy sources can sustain continuous operation in remote agricultural environments, providing essential data for farmers.

Efficiency in data collection and processing is paramount. Systems that utilize microcontrollers like ESP32, as highlighted by [27], allow for seamless integration with various sensors while maintaining low power consumption. This efficiency is crucial in field deployments, where energy resources may be limited.

The communication layer connects local stations to wider networks and platforms. Technologies such as Wi-Fi are employed for short-range, infrastructure-supported deployments [43,44], while LoRa/LoRaWAN are preferred for long-range, low-power applications, enabling reliable coverage in expansive or rural fields [35,58]. Cellular networks (GSM/GPRS) provide essential backhaul where other connectivity options are unavailable, though they introduce higher costs and energy demands [49,50]. Moreover, this is essential for capturing microclimatic variability across large fields, as demonstrated in the studies by [31].

The application and service layer integrates cloud platforms and visualization dashboards (e.g., ThingSpeak, mobile/web apps), which deliver real-time access, historical data trends, and decision-support tools for farmers [52]. These services enhance usability by translating sensor outputs into actionable insights for irrigation management, disease forecasting, and climate adaptation [59].

Reliability and standardization remain essential across all layers. As [60,61] emphasized, calibration of low-cost sensors and interoperability among hardware and software components are critical to ensuring data accuracy and comparability. Furthermore, autonomy is increasingly prioritized, with energy-efficient duty cycling and solar harvesting supporting long-term resilience of field-deployed stations [56,62].

In conclusion, the architecture of low-cost IoT agrometeorological systems reflects a balance between affordability, energy autonomy, and scalability. By combining modular sensing, efficient data processing, and layered communication frameworks, these systems provide actionable, real-time information that strengthens decision-making and resource management in climate-smart agriculture.

### 3.3. Applications in Climate-Smart Agriculture

The reviewed studies collectively demonstrate that low-cost IoT-enabled agrometeorological stations significantly contribute to climate-smart agricultural practices by enabling precise irrigation management, crop protection, and environmental risk assessment. Integrated soil and weather monitoring systems, such as those presented by [38,59], utilize predictive irrigation scheduling that not only optimizes water use up to almost 30% but also reduces labor demands, leading to improved resource efficiency and potential yield stability under water-limited conditions. By preventing over- or under-irrigation, these systems enhance soil health and crop resilience, which are key components of sustainable yield improvement [28].

Portable and modular stations, such as demonstrated by [63], further extend their utility by enabling localized microclimate assessments for frost protection and zoned irrigation. This capacity for targeted interventions safeguards sensitive crops and sustains productivity. Likewise, applications in disease risk modeling [31] and wind-based spray drift management [51], highlight the role of IoT stations in reducing crop losses due to pests and chemical misapplication, both of which have direct implications for yield and quality.

Studies by [30,55], have integrated real-time weather data with adaptive decision-support models to automate farm operations. Ref. [55] notably reported water savings of nearly 11% and improved orchard management through an adaptive Partial Least Squares (PLS) model, which dynamically predicted irrigation needs based on real-time soil and weather data. Such adaptive systems not only conserve water but also maintain optimal soil moisture levels, thereby enhancing crop growth and yield potential.

Energy-autonomous and GSM-enabled systems, as highlighted by [11,48], ensure uninterrupted operation in off-grid or rural settings, making precision farming accessible to smallholder farmers who otherwise face infrastructure limitations. This continuous data availability allows for proactive responses to environmental stressors such as heatwaves or frost events reducing potential yield losses due to climatic extremes.

The reviewed studies demonstrated that low-cost IoT agro-weather stations contribute to yield enhancement by optimizing irrigation schedules [38,55], mitigating disease and pest risks [31,51], and providing actionable real-time data for climate adaptation strategies. By integrating predictive modeling with localized monitoring, these systems ensure that crops receive the right amount of water, nutrients, and protection at the right time, leading to improved productivity and quality while reducing input costs and environmental impact.

Additionally, agrometeorological stations are most valuable were real-time, site-specific data drive day-to-day decisions. In cereal systems, Agrometeorological data (soil/air temperature, rainfall, and near-surface humidity) are used to time sowing and reduce emergence risk after adverse rain or cold spells [64]. In horticultural crops, continuous tracking of rainfall and reference evapotranspiration (ET_0_) combined with soil-moisture readings is central to irrigation scheduling, improving water-use efficiency and stabilizing yields [65]. In orchards and other perennial systems, station-derived microclimate indicators (leaf-wetness duration, temperature humidity profiles) underpin pest and disease forecasting, enabling targeted, earlier interventions. During harvest, short-term forecasts and on-site humidity, wind, and rainfall probabilities help select safe harvest windows to protect quality and limit post-harvest losses [66]. Integrating these data streams has consistently improved productivity, resource efficiency, and operational timing across diverse cropping systems.

### 3.4. Adaptability, Usability, and Scalability in Agricultural Technology

Adaptability emerges as a cornerstone of low-cost IoT-enabled agrometeorological stations, with numerous studies emphasizing modular, component-based designs that can be tailored to diverse agronomic contexts. Ref. [61] demonstrated a modular architecture using the EXMAN component model, enabling plug-and-play integration of varied sensors and communication protocols, thereby supporting rapid reconfiguration for different crops, climates, and farm scales. Similar adaptability is reflected in studies like [34,56], which incorporated multi-sensor suites (e.g., soil moisture, solar radiation, and air quality) and modular communication (LoRa, WiFi) to meet site-specific monitoring requirements. Field application examples, such as the implementation of these modular systems in community farms, have shown that farmers can easily adapt the technology to different crops, resulting in improved water management and increased yields. User feedback from these implementations has indicated a strong appreciation for the flexibility and ease of use of these systems, with farmers reporting enhanced decision-making capabilities. Such flexibility aligns with findings by [67,68], which stress that modular designs ensure the longevity and upgradability of IoT platforms in agricultural settings.

Usability is enhanced through intuitive user interfaces, mobile applications, and cloud dashboards, enabling real-time monitoring, automated alerts, and historical trend analysis. Refs. [69,70] showcased systems that integrate smartphone-based dashboards with lightweight MQTT protocols, empowering even non-technical users to interpret and act on environmental data. In practical applications, user feedback has highlighted that farmers find these mobile interfaces accessible and useful for making timely decisions regarding irrigation and pest management. For example, a survey conducted among users of an integrated IoT platform revealed that over 80% of farmers reported improved engagement with their monitoring systems due to user-friendly interfaces. Ref. [31] further reinforced usability by implementing multi-channel visualization platforms including Android apps, websites, and ThingSpeak dashboards thus improving farmer engagement and data accessibility. Ref. [30] introduced fuzzy logic-based decision support that translated environmental metrics into actionable alerts (e.g., heat stress or disease risk), demonstrating how usability can be enhanced through context-aware data interpretation.

Scalability is driven by open-source hardware and software, along with cost-effective components, which allow dense network deployments across large agricultural areas [71]. Ref. [36] highlighted a participatory, design-based learning approach, where high-school students built reliable weather data systems that contributed to community-driven weather intelligence networks. This initiative not only demonstrated the adaptability of the technology but also involved farmers in the design process, leading to increased acceptance and adoption of these systems in their operations. Likewise, ref. [35] demonstrated a scalable LoRaWAN-enabled network of 25 stations across urban landscapes, which offers a transferable blueprint for high-density farm deployments, ensuring improved microclimatic coverage and data resolution. Complementary studies, such as [29,72], emphasize that open-source frameworks and interoperability standards are critical to maintaining scalability, data quality, and integration across heterogeneous agricultural monitoring systems. In this context, it is also essential to consider the digitization footprint: because IoT depends on continuous data exchange, the quantity, frequency, and type of data collected directly shape bandwidth, storage, and energy demands [72]. Stations measuring core climatic variables (temperature, humidity, rainfall) impose a modest digital burden, whereas imaging-enabled systems generate substantially larger datasets requiring more transmission and processing resources. While richer data can improve decision support, they raise questions of energy use, infrastructure sustainability, and long-term maintainability [73]. Balancing scalability gains with an appropriate digitization footprint ensures IoT weather networks remain agronomically effective, operationally viable, and environmentally sustainable.

### 3.5. Evaluation of Cost, Accuracy, and Reliability Metrics

The evaluation of IoT-enabled agrometeorological stations reveals important trade-offs among cost, accuracy, and reliability. Studies highlight a wide range of system designs, from low-cost prototypes to more advanced, high-performance deployments.

A bespoke monitoring network was investigated in [35], using LoRaWAN technology for urban heat island assessment. While specific costs were not detailed, the study highlighted the use of high-performance sensors with accuracies of ±0.1 °C for temperature and ±1.5% to ±2.0% for humidity, offering high reliability through long-range, low-power data transmission.

In contrast, an Arduino-based portable weather monitoring system was presented in [48] with an approximate cost of $98. This system-maintained measurement accuracy within 5% of conventional devices and demonstrated an estimated power consumption of 1–2 watts, emphasizing its suitability for remote monitoring.

Similarly, a low-cost, solar-powered weather station for agricultural use was developed by [56], with a total cost of approximately $146.23. The system showed strong correlations to reference stations with R^2^ values exceeding 0.99 for temperature, 0.98 for humidity, and 0.99 for rainfall, and it operated reliably for up to 17 h without sunlight on a 3000 mAh battery. This design illustrates how energy autonomy can be paired with high accuracy at relatively low cost.

A retracted study [58] also explored IoT-driven model for precision irrigation using machine learning. The study noted a prediction accuracy of 91.25% with the Linear Discriminant Analysis algorithm, though details on cost and reliability were not specified.

Taken together, these studies collectively underscore the diverse approaches and innovations in the field, highlighting the balance between affordability, precision, and operational efficiency in IoT-enabled weather monitoring systems.

## 4. Conclusions

This systematic review highlights the growing potential of low-cost IoT-enabled agrometeorological stations as transformative tools for climate-smart agriculture. By integrating cost-effective microcontrollers, open-source sensors, and flexible communication protocols such as WiFi, LoRaWAN, and GSM, these systems provide localized, real-time environmental data that support precise irrigation scheduling, disease and pest management, and adaptive responses to extreme weather events. Studies such as those by [31,38,55] demonstrated that these technologies can enhance water-use efficiency, optimize labor, and safeguard yields, particularly in resource-constrained or rural settings where conventional weather data systems are prohibitively expensive or inaccessible. Furthermore, modular, and open-source designs [35,60] enable scalability, ensuring that these solutions can be adapted to diverse agricultural contexts and expanded across larger landscapes or community-based networks.

Despite these advancements, several challenges remain. A persistent concern is the reliability and calibration of low-cost sensors, which, if unaddressed, can compromise data accuracy and decision-making [34]. Connectivity issues, especially in rural areas with limited infrastructure, and the lack of standardized protocols for data interoperability further hinder widespread adoption [20,73]. Additionally, many studies rely on prototype-level systems without conducting long-term performance evaluations under varying climatic and soil conditions, limiting their practical applicability. Equally important, issues of usability such as the need for intuitive mobile dashboards and farmer training remain barriers to adoption, as highlighted in the review’s analysis of adaptability and usability.

Despite the advancements outlined in this review, several challenges remain that necessitate further research. Future investigations should prioritize enhancing sensor reliability through the development of robust calibration protocols to improve the accuracy of low-cost sensors, thereby mitigating issues related to data reliability and ensuring that the information provided to farmers is trustworthy. Efforts must also be made to establish standardized protocols for data interoperability among different IoT systems, facilitating seamless integration of various components and enhancing the scalability of agrometeorological stations. Expanding on recent findings, future research should also explore hybrid communication architectures (combining Wi-Fi for local logging with LoRaWAN for remote telemetry) and energy-autonomous designs that integrate duty-cycling and solar harvesting, both of which are crucial for long-term deploy-ability. Additionally, the integration of advanced analytics, including AI-driven forecasting and adaptive decision-support models, could significantly improve predictive capabilities and optimize resource allocation in agricultural practices, enabling farmers to make more informed decisions based on real-time data. Conducting longitudinal field trials is essential to validate the agronomic and economic impacts of these systems, particularly their influence on yield, water productivity, and resilience to climate variability under different environmental conditions. Moreover, addressing the need for effective training programs will enhance farmer understanding and engagement with these technologies. Developing intuitive user interfaces and educational resources will empower smallholder farmers to fully utilize IoT-enabled agrometeorological systems. Lastly, strengthening collaborations between researchers, technology developers, and agricultural stakeholders will be critical in translating these innovations into scalable, farmer-friendly solutions that enhance climate resilience and support sustainable food production in vulnerable regions.

## Figures and Tables

**Table 1:** The recent systematic reviews regarding weather stations.

No	Article’s Title	Key Findings	Limitations	References	Year
1	Weathering the storm: A systematic literature review of weather stations’ observational practices	The review finds that weather observations in the Cordillera Administrative Region are generally accurate, with occasional errors caused by instrument calibration issues and human factors, especially during extreme weather events. While errors are infrequent, they tend to increase under high workloads and during rapid decision-making. Improving training, stress management, and quality control systems is essential to maintain data reliability. Additionally, adopting low-cost technologies and integrating local knowledge can enhance coverage and forecast relevance, particularly in resource-limited areas.	Calibration, maintenance, and human recording errors (worse during extreme events). Data gaps from equipment, power, communication failures. Findings are context specific to PAGASA CAR stations (limited generalizability).	[17]	2024
2	Weather Forecasting: A Systematic Review Using AI Approaches	This review underscores the significant contribution of artificial intelligence, particularly machine learning and deep learning models, in enhancing the accuracy of weather forecasting through efficient analysis of extensive and complex datasets. The study highlights the increasing importance of AI in critical sectors including agriculture, energy, and disaster management, with future developments anticipated through hybrid approaches and quantum models.	Region- and range-specific datasets result in variable model performance, with limited standardized benchmarks and inadequate uncertainty quantification. The ability to generalize beyond studied regions remains unclear, while model reliability is strongly constrained by the quality of historical data. Furthermore, challenges persist in model interpretability and in achieving robust validation under real-time and extreme event conditions.	[18]	2024
3	A review of weather conditions monitoring system based on IoT	This review highlights that IoT-based weather monitoring systems utilizing Arduino platforms are effective for real-time climate data collection, including parameters such as temperature, humidity, atmospheric pressure, and precipitation. These systems offer benefits such as remote monitoring, data logging, graphical visualization, and real-time alert capabilities, making them suitable for applications in agriculture, environmental research, and weather forecasting. The review also notes certain limitations, including dependency on internet connectivity, lack of gas sensors, and limited data storage solutions. Overall, Arduino-based IoT systems present a cost-effective and promising approach for weather measurement, though enhancements in system reliability and sensor integration are recommended to support broader, long-term applications.	IoT platforms and sensors face challenges such as limited standardization and calibration comparability, dependence on stable power supply and internet connectivity, insufficient long-term real-world validation, gaps in data management and security, and a lack of comprehensive assessment of cost and scalability.	[19]	2024
4	The impact of IoT and sensor integration on real-time weather monitoring systems: A systematic review	The review indicates that integrating smart, real-time weather monitoring systems with mobile applications utilizing technologies such as IoT, Arduino, sensors, machine learning, and cloud computing has significantly improved the accuracy and accessibility of weather forecasts. These systems facilitate real-time data collection of key weather parameters, including temperature, humidity, and wind speed, thereby supporting more informed decision-making across various sectors such as agriculture, transportation, aviation, and disaster management. The application of machine learning enhances predictive accuracy, while cloud integration provides secure, remote access to data. Overall, the findings suggest that IoT-based weather monitoring systems have the potential to fundamentally improve the way weather information is gathered, analyzed, and utilized.	Sensor accuracy issues caused by environmental factors, high energy use, and connectivity problems. Lack of standardization, high costs of advanced sensors, and challenges in real-time data transmission and storage also hinder implementation. Additionally, maintenance and durability in field conditions remain significant concerns.	[14]	2023
5	The contribution of weather forecast information to agriculture, water, and energy sectors in East and West Africa: A systematic review	The findings indicate that weather information services in East and West Africa predominantly serve the agricultural sector and are primarily accessed through radio, mobile phones, and television. However, their effectiveness is constrained using generic forecasts, communication challenges, a lack of tailored and localized information, and limited user capacity. These issues underscore the necessity for enhanced stakeholder training and the development of more targeted and context-specific climate information dissemination strategies.	The review shows that weather forecasts mainly support agriculture, with less focus on water and energy. Forecasts are mostly rainfall and temperature, delivered via radio, TV, and mobile phones. Limitations include being generic rather than impact-based, poor communication, lack of local detail, low trust, and reliance on indigenous knowledge. Capacity-building is needed to improve uptake and use across sectors.	[20]	2022
6	Technological opportunities for sensing of the health effects of weather and climate change: a state-of-the-art-review	The report highlights the importance of establishing standardized measurement protocols, promoting data transparency, and ensuring ethical management of personal data collected through wearable technologies. It also advocates for interdisciplinary collaboration and open science to address fragmented research efforts and resource constraints. Overall, integrating advanced sensing technologies with cross-sectoral data and fostering inclusive global partnerships are critical for advancing biometeorological research and effectively addressing climate-related health risks.	Current sensing technologies face challenges of high cost, limited accessibility in low-resource settings, and difficulties in integrating heterogeneous data from multiple sensors. Many systems lack standardization, making interoperability and large-scale deployment difficult. Data privacy and security concerns further limit adoption, while long-term reliability and maintenance of sensors in diverse environments remain problematic. Additionally, there is a shortage of studies linking sensor data directly to measurable health outcomes, creating gaps between technology development and practical health applications.	[21]	2021
7	Extreme weather events in agriculture: A systematic review	This review highlights the expanding global research on extreme weather events (EWE) in agriculture, primarily focusing on staple crops such as wheat, maize, and rice. Notable gaps include a limited number of studies on high-value crops (e.g., tomatoes, grapevines), underutilization of remote sensing technologies, and insufficient emphasis on mitigation strategies and governance, particularly in vulnerable developing countries (e.g., USA, UK). Additionally, the review underscores disparities in international collaboration, with developed nation’s leading research efforts. Addressing these gaps is essential for enhancing climate resilience in the agricultural sector.	The review notes gaps in remote sensing for monitoring extreme weather impacts, limited studies on high-value crops and developing countries, and a lack of research on mitigation strategies and governance frameworks.	[22]	2019
8	Weather monitoring station: a review	The review outlines that wireless weather monitoring systems utilizing microcontrollers such as ARM, PIC, and AVR provide faster data processing, lower power consumption, and decreased need for manual intervention. These systems facilitate real-time, remote monitoring of weather conditions through technologies such as GSM, Zigbee, and Radio Frequency, offering cost-effective and scalable alternatives to traditional manual and wired approaches.	Key issues include the high cost and complexity of some systems, limited accuracy of low-cost sensors, and challenges with data transmission and storage in real-time applications. The study also notes problems with maintenance, calibration, and durability of sensors in outdoor environments, as well as the lack of standardization in system design, which makes scalability and integration difficult.	[23]	2016

**Table 2:** Application of the SPIDER framework.

Component	Review Content
**S—Sample**	Weather stations or monitoring systems using IoT (possibly in rural/urban deployments)
**PI—Phenomenon of Interest**	Use of IoT-based low-cost weather stations to monitor environmental parameters
**D—Design**	Technical case studies, field experiments, deployment trials, performance assessments
**E—Evaluation**	Effectiveness, accuracy, data transmission reliability, cost-efficiency, deployment challenges
**R—Research type**	Mixed methods (quantitative data on performance, qualitative insights on implementation)

## Data Availability

Data available upon request.
